# Vaping, an emerging public health concern in South Asia: a short communication

**DOI:** 10.1097/MS9.0000000000000297

**Published:** 2023-03-27

**Authors:** Fahad Gul, Alishba A. Khan, Syed N.H. Kazmi, Khawar Abbas, Jawad Basit

**Affiliations:** Departments of a Medicine; b Surgery, Rawalpindi Medical University, Rawalpindi, Pakistan

**Keywords:** Vaping, electronic cigarettes, smoking cessation, nicotine

## Abstract

Electronic cigarettes, or “vaping,” are battery-operated devices that heat a liquid containing propylene glycol, nicotine, and some flavoring agents, which aerosolize to produce vapors that the user inhales. They were introduced in 2003 and became popular worldwide as a less irritating alternative to combustible cigarettes. While they were initially advertised as smoking cessation aids, their use has taken the shape of an epidemic in some regions of the world. Vaping prevalence is high in South Asia, where a significant number of people use tobacco and smokeless tobacco. According to data from Pakistan, 6.2% of the population uses vaping/e-cigarettes, while 15.9 million (12.4%) use smokeless tobacco. Vaping may be a safer alternative to cigarette smoking, as e-cigarettes do not contain all the toxins that regular cigarettes do, and the aerosol from e-cigarettes has no appreciable cytotoxic, genotoxic, or inflammatory effects when inhaled. However, nicotine addiction is a concern, as it is the main culprit behind smoking addiction, and e-cigarettes may become a new pathway toward nicotine addiction. Hence, their effectiveness in smoking cessation is still debatable, and their role as a tool for smoking cessation needs further research.

## Introduction

Over the past few decades, there has been a significant surge in the usage of electronic cigarettes, popularly known as ‘vaping’^1^. Electronic cigarettes operate on a battery that heats a liquid containing propylene glycol, nicotine, and some flavoring agents, thus aerosolizing it to produce a vapour, which is then inhaled by the user. They contain a nicotine-laced fluid with some flavors and preservatives, which is smoked by the user^2^. The whole idea is to provide nicotine to users with less irritation than combustible cigarettes. Electronic cigarettes were introduced first in 2003 by a Chinese inventor and made their way to Europe and North America by 2006. They were first advertised as a smoking cessation aid, but their use quickly reached epidemic levels in some regions of the world^3^. Data suggest that the characteristics of Electronic Nicotine Delivery Systems products result in inhalational toxicity^4^.

## Prevalence of vaping in south Asia

Smoking cessation is the most important factor and the first step toward halting the effects of smoking. Some smoking cessation pills are carcinogenic, so most people switch to Electronic Nicotine Delivery Systems as an alternative. In 2015, a survey of 121 countries concluded that 351.9 million people use smokeless tobacco (SLT), and around 90% of them live in South Asia^4^. According to data for the subcontinent, 35% of Bangladeshis and 29% of Indian adults, respectively, use tobacco in some capacity. In Pakistan, 23.9 million individuals (19.1%) smoke regularly, according to statistics. Around 6.2% of the population uses vaping or e-cigarettes, while 15.9 million (12.4%) use SLT in the form of Naswar^4^. In another study carried out in Sindh, Pakistan, about 65.6% of participants claimed to be aware of electronic cigarettes, while 6.2% claimed to be using them. In comparison to nontobacco users, persons who use conventional tobacco have a greater understanding of vaping^5^. SLT consumption among Pakistani females is lower than among Bangladeshi (27.9%) and Indian (18.4%) women. Table 1 shows a comparison of the use of tobacco-based products among countries in the subcontinent. E-cigarettes and vaping were outlawed in India in 2019, but sales increased by almost twice that amount, from 1.6 to 3.3 million between 2014 and 2019. Bangladesh likewise intended to outlaw electronic cigarettes; however, the number of users is unpopular. E-cigarettes and vaping were outlawed in India in 2019, yet despite this, sales more than doubled from 1.6 to 3.3 million between 2014 and 2019, nearly tripling. Bangladesh also intended to outlaw electronic cigarettes, but the numbers showing how many people use them are controversial^4^.

**Table 1:** Use of tobacco-based products in South Asia.

Country	Tobacco	Smokeless tobacco	Vaping/E-cigarettes	Total
Pakistan	23.9 million (19.1%)	15.6 million (12.4%)	6.2%	9.6 million (7.7%)
India	99.5 million (10.7%)	199.4 million (21.4%)	268 000 0.02%	266.8 million (28.6%)
Bangladesh	19.2 million (18.2%)	22 million (20.6%)	0.2%	37.8 million (35.3%)

## Is vaping a safer alternative to cigarette smoking?

E-cigarettes offer an advantage because they do not contain all the toxins that regular cigarettes do, making them a safer alternative for those who want to quit smoking^6^. Nicotine does not cause cancer on its own, and the aerosol from e-cigarettes has no appreciable cytotoxic, genotoxic, or inflammatory effects when inhaled^7^. Many studies have demonstrated that e-cigarettes carry a lesser health risk than conventional cigarettes, whose ill effects on all human body systems have been well established, and produce less passive smoke ,thus reducing environmental hazards as well. The other clinical advantage that is claimed by companies is that e-cigarettes help with smoking cessation. Many chronic smokers who wish to discontinue resort to e-cigarettes as they are a safer alternative. The efficacy of e-cigarettes in helping smokers quit is still debatable. Longitudinal and cross-sectional studies smoke have reported an increase in abstinence or smoking cessation among those who use e-cigarettes^8^-^10^. However, a meta-analysis that evaluated the role of e-cigarettes in smoking cessation reported that individuals who use e-cigarettes have 28% lower odds of smoking cessation compared to those who do not smoke e-cigarettes^11^. Hence, the effectiveness of e-cigarettes in smoking cessation is still not proven. It is still not established whether e-cigarettes should be promoted as tools for smoking cessation.

One disadvantage is the development of nicotine addiction in users of electronic cigarettes, as nicotine is the main culprit behind smoking addiction as well. Nicotine is known to be a gateway drug leading to the development of addiction to other addictive substances, and this can be a concern with the emerging use of e-cigarettes, which can become a new pathway toward nicotine addiction. Nonsmokers may start experimenting with e-cigarettes, become addicted to nicotine, and then turn to smoking conventional cigarettes to feed their nicotine addiction. Dual usage of both e-cigarettes and conventional cigarettes has been reported in some studies^12^,^13^. Highlighting the gateway potential of nicotine, a few observational studies have also reported concurrent cannabis usage in e-cigarette users. E-cigarettes have also become a popular mode of delivering cannabis products^14^. Hence, marketing e-cigarettes as a safer alternative can be misleading.

There have been multiple reports of e-cigarette or vaping product-associated lung injuries in adolescents, with many patients being hospitalized in ICUs and requiring mechanical ventilation. In 2019, 2600 cases and 68 deaths were reported in the United States, with the majority of the cases being young and adolescent teenagers^15^. The pathogenesis of e-cigarette or vaping product-associated lung injuries is still not well understood but plausible mechanisms include vitamin E acetate and tetrahydrocannabinol as the culprits. Vitamin E acetate may alter the lung surfactant function and hence cause respiratory dysfunction^16^. E-cigarettes may also contain some hazardous compounds formed as a result of heating the liquid, which include formaldehyde, acetaldehyde, and acrolein, which are known to be cytotoxic^17^. Vaporization may also cause the release of a trace amount of metals such as tin, nickel, and cadmium but it is not documented whether these metals are present in an unsafe quantity^18^.

## Recommendation

In the South Asian vaping revolt, it is crucial to solve the numerous flaws of present treaties, similar to how the WHO’s Framework Convention on Tobacco Control, was originally negotiated and came into force in 2005. The Global Youth Tobacco Survey which is supported by the WHO, is advised to be used as a starting point for data collection. The socioeconomic inequalities among consumers will be better understood with more data gathered from various South Asian cities. Hence, better policies will be implemented.

The many marketing techniques used in the vaping industry must be kept up-to-date by decision makers. A life-course approach should be used to target consumers in visible and strong antitobacco efforts. This intervention must be planned to use recently compiled data, which must be tailored to the Global Youth Tobacco Survey or other reliable data collection methods. Before the person turns 18, the behavioral intervention components may be included to increase compliance. This can be done by examining school-based programmes and strictly enforcing laws regarding tobacco access for those under the age of 18. Conversely, tobacco prevention programmes for people over 18 must target those with low socioeconomic status and a low level of education.

An established and affordable strategy for tobacco control and reducing the harmful health and economic effects of tobacco use is raising taxes on e-cigarettes and tobacco-based products. The WHO recommends a uniform, specific excise tax that accounts for at least 75% of the retail price as a practical way to achieve this. By imposing financial penalties on first-time and repeat violators, it would also aid in the enforcement of already-existing rules and regulations.

## Conclusion

There is no conclusive or strong evidence for any beneficial outcome from e-cigarettes. The effectiveness of e-cigarettes in helping smokers quit is still not proven. E-cigarettes might help some individuals stop smoking. So they should only be available via prescription from authorized medical professionals trained in helping people quit. Any access beyond these risks serious harm for no benefit.

## Ethical approval

Not applicable.

## Consent

Not required.

## Sources of funding

No funding is required for the study

## Author contribution

F.G., A.A.K., and S.N.H.K.: conception, write-up, critical review, and approval of the final version. K.A. and J.B.: write-up, critical review, and approval of the final version.

## Conflicts of interest disclosure

All authors declared no conflict of interest.

## Research registration unique identifying number (UIN)

1. Name of the registry: not applicable.
2. Unique identifying number or registration ID: not applicable.
3. Hyperlink to your specific registration (must be publicly accessible and will be checked): not applicable.

## Provenance and peer review

Not commissioned, externally peer reviewed.
